# Potential of Anti-Leukotriene Drugs as New Therapeutic Agents for Inhibiting Cholangiocarcinoma Progression

**DOI:** 10.3390/molecules29143379

**Published:** 2024-07-18

**Authors:** Yusuke Kito, Kenta Kachi, Michihiro Yoshida, Yasuki Hori, Akihisa Kato, Hidenori Sahashi, Tadashi Toyohara, Kayoko Kuno, Akihisa Adachi, Kenji Urakabe, Hiromi Kataoka

**Affiliations:** 1 Department of Gastroenterology and Metabolism, Graduate School of Medical Sciences, Nagoya City University, Nagoya 467-8601, Japan; oll3a@med.nagoya-cu.ac.jp (Y.K.); mityoshi@med.nagoya-cu.ac.jp (M.Y.); yhori@med.nagoya-cu.ac.jp (Y.H.); akihisa@med.nagoya-cu.ac.jp (A.K.); hsahashi@med.nagoya-cu.ac.jp (H.S.); toyohara@med.nagoya-cu.ac.jp (T.T.); kayopon306024@yahoo.co.jp (K.K.); aada0313@med.nagoya-cu.ac.jp (A.A.); open98percent@gmail.com (K.U.); hkataoka@med.nagoya-cu.ac.jp (H.K.); 2 Department of Gastroenterology, Gifu Prefectural Tajimi Hospital, Tajimi 507-8522, Japan

**Keywords:** cholangiocarcinoma, montelukast, zileuton, drug repositioning, cysteinyl leukotriene receptor 1, leukotriene D_4_

## Abstract

Cholangiocarcinoma (CCA) is a cancer with a poor prognosis due to difficulties in diagnosis and limited treatment options, highlighting the urgent need for new targeted therapies. In a clinical setting, we found that leukotriene levels in bile were higher than in serum. Immunohistochemical analysis of surgically resected samples also revealed that CysLT receptor 1 (CysLTR1) was more highly expressed in CCA than in normal bile duct tissue, prompting us to investigate leukotriene as a potential therapeutic target in CCA. In vitro studies using CCA cell lines expressing CysLTR1 showed that leukotriene D4, a major ligand of CysLTR1, promoted cell proliferation, with increased phosphorylation of AKT and extracellular signal-regulated kinase 1/2 (ERK1/2). Additionally, treatment with two clinically available anti-allergic drugs—zileuton, an inhibitor of CysLT formation, and montelukast, a CysLTR1 inhibitor—had inhibitory effects on cell proliferation and migratory capacity, accompanied by the reduced phosphorylation of AKT and ERK1/2. Furthermore, the simultaneous administration of both drugs synergistically enhanced the inhibitory effect on cell proliferation. Our study suggests that use of these drugs may represent a novel approach to treat CCA through drug repositioning.

## 1. Introduction

Cholangiocarcinoma (CCA) is the second most common primary liver malignancy, accounting for 10–20% of newly diagnosed liver cancers with biliary features [1,2]. Unfortunately, most CCAs are diagnosed at an advanced stage and require systemic chemotherapy (with gemcitabine as the key drug) instead of surgery [2,3]. However, conventional chemotherapy strategies based on gemcitabine are often ineffective because of the early development of drug resistance. The efficacy of newer therapies such as immune checkpoint inhibitors and molecularly targeted drugs has recently been reported, but problems remain, including treatment limitations due to comorbidities and characteristic side effects (e.g., irAE) that are sometimes fatal [4]. Therefore, new approaches to the treatment of CCA are required.

Drug repositioning (DR), the study of existing drugs for new therapeutic purposes, is one of the most promising avenues to identify new preventive or therapeutic agents. DR can provide effective drugs quickly and at low cost because these agents have already passed clinical trials and have shown no safety or pharmacokinetic issues [5,6,7]. In recent years, DR has been extensively studied in the field of cancer treatment, one of the most significant challenges for medical professionals [8,9,10].

Among various drugs that have attracted attention as targets of DR in cancer treatment, we focused on anti-leukotriene drugs because there were papers reporting their anti-tumor effects based on large-scale epidemiological studies [11,12]. Recent epidemiological studies have shown that patients using cysteinyl leukotriene receptor antagonists (LTRAs) for asthma have a significantly lower risk of several cancers, including lung, breast, colon, and liver cancers, and this decrease is dose-dependent [11]. Additionally, a systematic review has summarized the association between various cancer types and leukotrienes [13]. Our group has previously focused on cysteinyl leukotrienes (CysLTs). We found that leukotriene D_4_ (LTD_4_), a type of CysLT, promotes the growth of pancreatic cancer cell lines and that montelukast, a type of LTRA, has anti-tumor effects in pancreatic carcinogenesis and pancreatic cancer cell lines [14]. However, there are few reports on the association between the development of CCA and anti-allergic drugs [13]. We focused on the fact that CysLTs have both renal and biliary excretion pathways. Thus, we hypothesized that if the relationship between biliary leukotrienes and CCA progression could be clarified, anti-leukotriene drugs could be proposed as new anti-tumor agents against CCA. Anti-leukotriene drugs currently in clinical use worldwide can be divided into two mechanisms of action, namely LTRAs and leukotriene synthesis inhibitors. Montelukast, which specifically targets CysLTR1, is one of the most important LTRAs currently in clinical use. Additionally, zileuton, a 5-lipoxygenase inhibitor, is the only agent in clinical use that inhibits leukotriene synthesis. Both of these drugs have been in clinical use for decades, and are relatively inexpensive and safe. From a DR perspective, it would be very significant if the efficacy of these drugs could be demonstrated in cholangiocarcinoma.

In the present study, we first measured CysLT concentrations in clinical samples of human bile and serum to determine their distribution in the body. We also analyzed the expression of CysLT receptor 1 (CysLTR1) in normal bile ducts and cancer sites using surgical specimens from patients with biliary tract cancer. Additionally, in vitro experiments were conducted to determine whether CCA cell lines express CysLTR1 and exhibit sensitivity to CysLTs. Furthermore, we investigated whether zileuton and montelukast have a preventive effect on cancer progression when applied to CCA cell lines alone or in combination.

## 2. Results

### 2.1. Clinical Characteristics of CysLTs and CysLTR1

**Figure 1:**
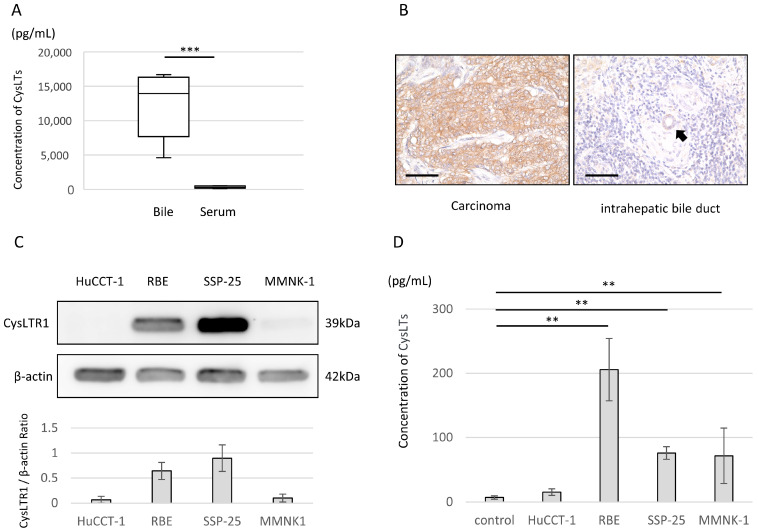
Characteristics of the clinical samples (bile and serum), pathological specimens, and cell lines used in the experiment. (**A**) Concentrations of CysLTs in bile and serum measured using the CysLT Express EIA Kit. Bars indicate standard deviation. ** *p* < 0.01, and *** *p* < 0.001. (**B**) Representative images of immunohistochemical staining for CysLTR1 expression in pathological specimens of intrahepatic cholangiocarcinoma. The black arrow indicates a normal intrahepatic bile duct. Magnification: ×400. (**C**) CysLTR1 expression in human CCA cell lines (HuCCT-1, RBE, and SSP-25) and the human immortalized cholangiocyte cell line MMNK-1. CysLTR1 levels were normalized against β-actin and represented the means of three independent experiments. (**D**) The amount of CysLTs in the supernatant during cell culture after 72 h of incubation.

Using clinical specimens, we analyzed the levels of CysLTs in bile and serum and immunohistochemically stained CCA tissues for CysLTR1, one of their receptors. The mean CysLT level was significantly higher in bile than in serum (12,442 ± 4360 vs. 310 ± 153 pg/mL, *p*  <  0.001) (Figure 1A). Immunohistochemical analyses showed that CysLTR1 was strongly expressed on the membrane of intrahepatic CCA cells, while normal intrahepatic bile duct epithelial cells did not express CysLTR1 (Figure 1B). Of the 10 CCA surgical specimens analyzed, 7 showed strong CysLTR1 expression at the carcinoma site. The clinical characteristics of the patients included in these experiments are summarized in Supplementary Tables S1 and S2.

### 2.2. CysLTR1 Expression and Synthetic Potential of CysLTs in Human CCA Cell Lines and the Human Immortalized Cholangiocyte Cell Line

Western blotting was performed to investigate the expression of CysLTR1 in human CCA cell lines and a human cholangiocyte cell line. Among the human CCA cell lines, CysLTR1 was highly expressed in RBE and SSP-25. By contrast, CysLTR1 was not expressed in the human CCA cell line HuCCT-1 or the human immortalized CCA cell line MMNK-1 (Figure 1C). An enzyme-linked immunosorbent assay (ELISA) was then carried out to determine the CysLT synthesis capacity of each cell line. After incubating these cell lines for 72 h, RBE (205.8 ± 48.6 pg/mL, *p*  <  0.01), SSP-25 (76.1 ± 9.8 pg/mL, *p*  <  0.01), and MMNK-1 (71.7 ± 43.0 pg/mL, *p*  <  0.01) synthesized and released CysLTs into the supernatant (Figure 1D).

### 2.3. LTD_4_ Contributed to Proliferation via CysLTR1 by Phosphorylating AKT and Extracellular Signal-Regulated Kinase 1/2 (ERK1/2) in Human CCA Cell Lines with the Receptor

**Figure 2:**
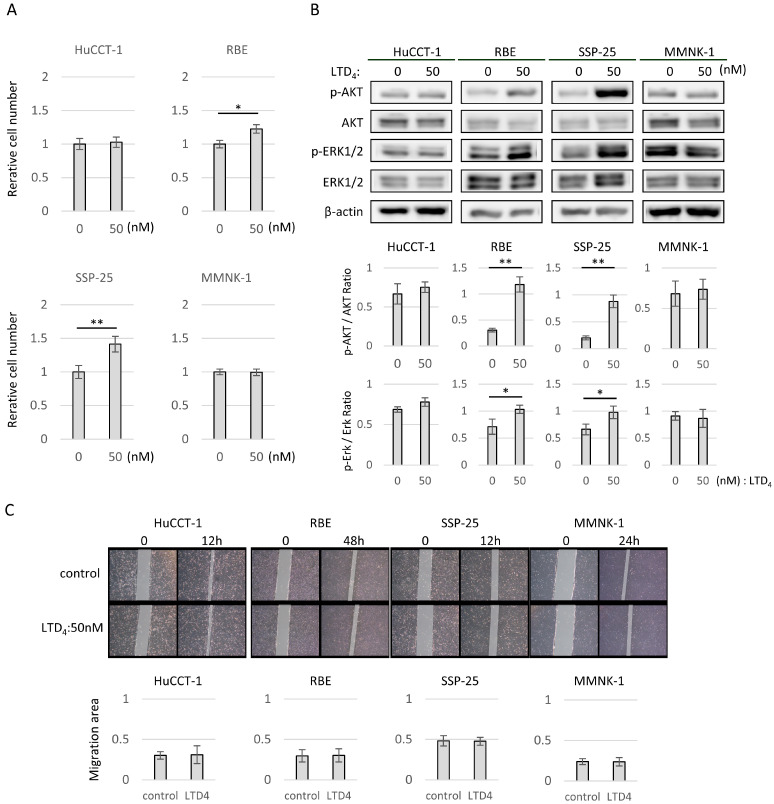
Effects of LTD_4_ on cell proliferation, migration, and intracellular signaling in CCA and cholangiocyte cell lines. (**A**) Bar graphs of relative cell numbers compared with the control at 72 h quantified by Cell Counting Kit-8 assay in low-CYSLTR1-expressing cell lines (HuCCT-1 and MMNK-1) and high-CYSLTR1-expressing cell lines (RBE and SSP-25). Cells were treated with or without LTD_4_ at 50 nmol/L. Data are presented as mean ± standard deviation *(n* = 3 per group). Bars indicate standard deviation. * *p* < 0.05, and ** *p* < 0.01 compared with controls. (**B**) Western blotting for RBE and SSP-25 treated with LTD_4_ at 50 nmol/L for 3 h. P-AKT levels were normalized against AKT, and p-ERK1/2 levels were normalized against ERK1/2. These represented the means of three independent experiments. Bars indicate standard deviation. (**C**) Representative images obtained at 12, 24, or 48 h after a scratch wound was made in confluent monolayers of HuCCT-1, RBE, SSP-25, and MMNK-1 cells. After the scratch, 50 nmol/L of LTD_4_ was added. The migration rates were quantified by measuring the area of the injured region.

We performed in vitro experiments to elucidate the promoting effects of LTD_4_, the agonist with the highest affinity for CysLTR1, on CCA progression. A Cell Counting Kit-8 assay was used to measure changes in cell proliferative capacity due to LTD_4_. LTD_4_ accelerated the proliferation of RBE and SSP-25 cells, which highly express CysLTR1 (50 nmol/L LTD4 in RBE cells: 1.22 ± 0.06, *p*  <  0.05, in SSP-25: 1.41 ± 0.11, *p*  <  0.01). By contrast, HuCCT-1 and MMNK-1 cells, which do not express CysLTR1, showed no proliferative effects (Figure 2A). Western blotting analysis revealed the up-regulation of phosphorylated AKT (p-AKT) and phosphorylated ERK (p-ERK), key proteins in the PI3K/AKT/mTOR cascade and the mitogen-activated protein kinase (MAPK) cascade downstream of CysLTR1 signaling on CysLTR1 high-expressing cell lines by stimulation with LTD_4_ (control vs. 50 nmol/L LTD_4_ in RBE cells: p-AKT/AKT ratio: 0.30 ± 0.04 vs. 1.18 ± 0.15, *p*  <  0.01, p-ERK/ERK ratio: 0.71 ± 0.13 vs. 1.03 ± 0.08, *p*  <  0.05; in SSP-25 cells: p-AKT/AKT ratio: 0.20 ± 0.04 vs. 0.88 ± 0.11, *p*  <  0.01, p-ERK/ERK ratio: 0.66 ± 0.10 vs. 0.98 ± 0.11, *p*  <  0.05) (Figure 2B). We conducted a wound-healing assay to evaluate the effects of LTD_4_ on cell migration. However, LTD_4_ treatment (50 nmol/L) did not affect cell migration in any of the cell lines (Figure 2C).

### 2.4. Zileuton and Montelukast Inhibited the Proliferative and Migratory Ability of CCA Cells in a Concentration-Dependent Manner

**Figure 3:**
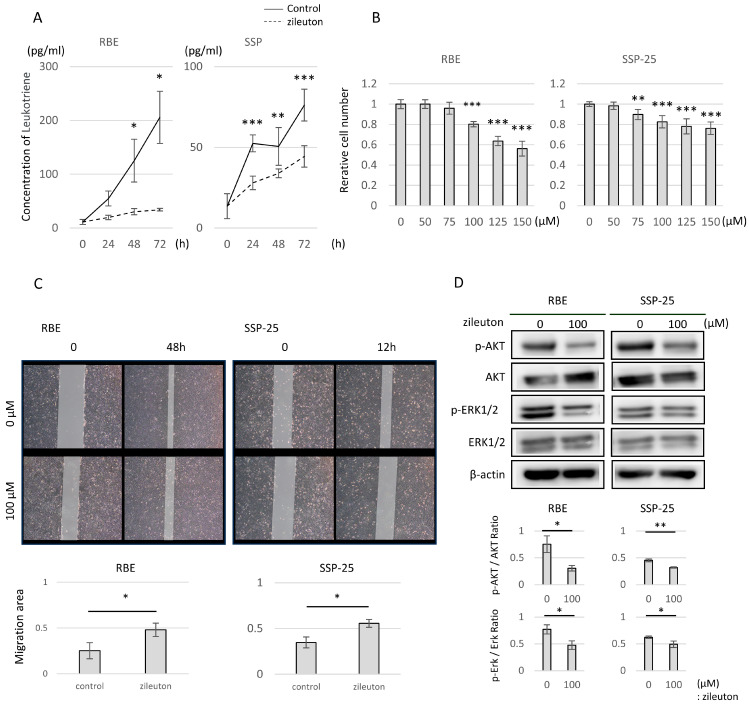
Effects of zileuton on cell proliferation, migration, and intracellular signaling. (**A**) The change in LTD_4_ concentration in the medium supernatant caused by zileuton administration was measured by a CysLTR1 ELISA kit. Data represent the means of four independent samples. Data are presented as mean ± standard deviation. * *p* < 0.05, ** *p* < 0.01, and *** *p* < 0.001. (**B**) Graphs of relative cell numbers compared with the control at 72 h quantified by a Cell Counting Kit-8 assay in high-CYSLTR1-expressing cell lines (RBE and SSP-25). Cells were treated with zileuton at 0–150 µmol/L. (**C**) Representative images obtained at 12 or 48 h after a scratch wound was made in confluent monolayers of RBE and SSP-25 cells. After the scratch, 50 µmol/L of zileuton was added. The migration rates were quantified by measuring the area of the injured region. (**D**) Western blotting for RBE and SSP-25 treated with zileuton at 100 µmol/L for 24 h. P-AKT levels were normalized against AKT, and p-ERK1/2 levels were normalized against ERK1/2. These represented the means of three independent experiments. Bars indicate standard deviation.

**Figure 4:**
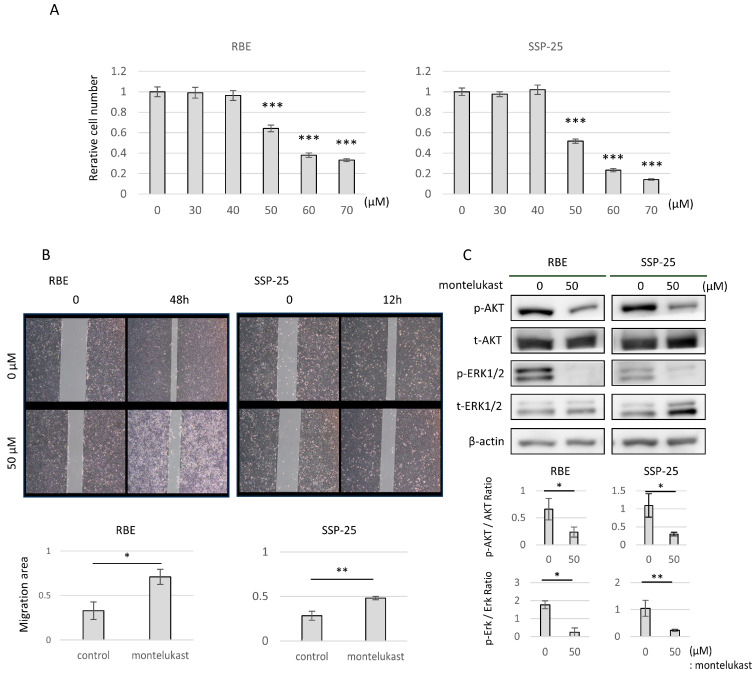
Effects of montelukast on cell proliferation, migration, and intracellular signaling. (**A**) Graphs of relative cell numbers compared with the control at 72 h, quantified by a Cell Counting Kit-8 assay in high-CYSLTR1-expressing cell lines (RBE and SSP-25). Cells were treated with montelukast at 30–70 µmol/L. Data are presented as mean ± standard deviation (*n* = 3 per group). Bars indicate standard deviation. * *p* < 0.05, ** *p* < 0.01, and *** *p* < 0.001 compared with control. (**B**) Representative images obtained at 12 or 48 h after a scratch wound was made in confluent monolayers of RBE and SSP-25 cells. After the scratch, 50 µmol/L of montelukast was added. The migration rates were quantified by measuring the area of the injured region. (**C**) Western blotting for RBE and SSP-25 treated with montelukast at 50 µmol/L for 24 h. P-AKT levels were normalized against AKT, and p-ERK1/2 levels were normalized against ERK1/2. These represented the means of three independent experiments. Bars indicate standard deviation.

Cell culture experiments were performed with zileuton (a 5-LOI) and montelukast (an LTRA) to determine their effects on CCA cells with CysLTR1. In the CCA cell lines RBE and SSP-25, LTD_4_ was synthesized and released into the supernatant during cell culture. However, 50 µM zileuton significantly reduced the concentration of LTD_4_ in the supernatant (50 nmol/L LTD_4_ vs. control in RBE at 48 h: 30.3 ± 5.8 vs. 125.1 ± 40.0 pg/mL, *p*  <  0.05; in SSP-25 at 24 h: 28.0 ± 4.2 vs. 52.5 ± 5.2 pg/mL, *p*  <  0.01) (Figure 3A). Both zileuton and montelukast inhibited cell proliferation in RBE and SSP-25 cells in a concentration-dependent manner (Figure 3B and Figure 4A). Zileuton significantly inhibited cell proliferation (100 µmol/L zileuton in RBE cells: 0.80 ± 0.02, *p* < 0.001, in SSP-25: 0.83 ± 0.07, *p* < 0.001), and montelukast significantly inhibited cell proliferation (50 µmol/L montelukast in RBE cells: 0.64 ± 0.03, *p* < 0.001, in SSP-25: 0.52 ± 0.02, *p* < 0.001). Next, when 100 µM zileuton or 50 µM montelukast was added to RBE and SSP-25 cultures, a reduction in cell migration capacity was observed after 48 h in RBE and 12 h in SSP-25 (Figure 3C and Figure 4B). Western blotting revealed that this suppression was caused by the down-regulation of the phosphorylation of AKT and ERK1/2 intracellular signaling relative to LTD_4_ treatment (control vs. 100 µmol/L zileuton in RBE cells: p-AKT/AKT ratio: 0.75 ± 0.30 vs. 0.31 ± 0.05, *p*  <  0.05, p-ERK/ERK ratio: 0.76 ± 0.08 vs. 0.48 ± 0.08, *p*  <  0.05; in SSP-25 cells: p-AKT/AKT ratio: 0.45 ± 0.03 vs. 0.32 ± 0.01, *p*  <  0.01, p-ERK/ERK ratio: 0.62 ± 0.02 vs. 0.49 ± 0.06, *p*  <  0.05; and control vs. 50 µmol/L montelukast in RBE cells: p-AKT/AKT ratio: 0.66 ± 0.20 vs. 0.23 ± 0.09, *p*  <  0.05, p-ERK/ERK ratio: 1.76 ± 0.21 vs. 0.24 ± 0.24, *p*  <  0.05; in SSP-25 cells: p-AKT/AKT ratio: 1.09 ± 0.32 vs. 0.30 ± 0.05, *p*  <  0.05, p-ERK/ERK ratio: 1.04 ± 0.29 vs. 0.23 ± 0.04, *p*  <  0.05) (Figure 3D and Figure 4C).

### 2.5. Co-Administration of Zileuton and Montelukast in Cell Culture Synergistically Enhanced the Inhibitory Effect on Cell Proliferation Which Was Mediated by Non-Apoptotic Cell Cycle Arrest

**Figure 5:**
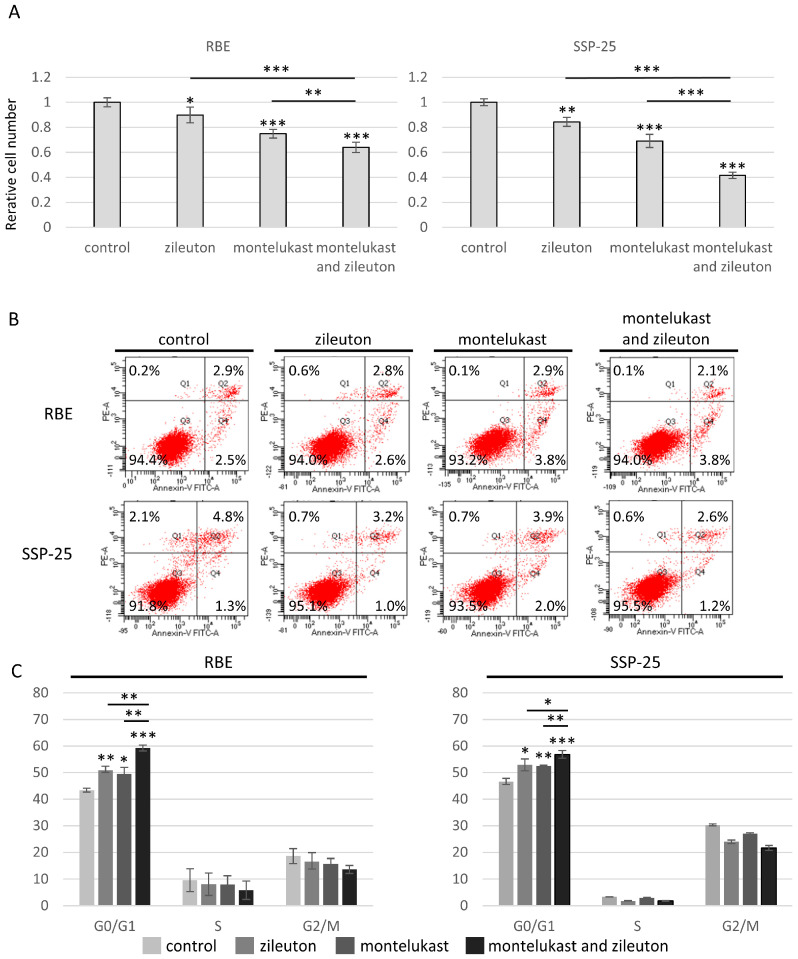
Effects of co-administration of zileuton and montelukast on CCA cell lines. (**A**) Graphs of relative cell numbers compared with the control at 72 h quantified by a Cell Counting Kit-8 assay in high-CYSLTR1-expressing cell lines (RBE and SSP-25). Cells were treated with zileuton at 100 µmol/L and/or montelukast at 50 µmol/L for 72 h. Data are presented as mean ± standard deviation (*n* = 3 per group). Bars indicate standard deviation. * *p* < 0.05, ** *p* < 0.01, and *** *p* < 0.001. (**B**) RBE and SSP-25 cells were treated with 100 µmol/L zileuton and/or 50 µmol/L montelukast for 72 h and then stained with annexin-V FITC/PI. Apoptotic cells were evaluated using flow cytometry. Data represent the means of three independent experiments. (**C**) RBE and SSP-25 cells were treated with 100 µmol/L zileuton and/or 50 µmol/L montelukast for 72 h, and cell cycles were determined using flow cytometry. Data are presented as mean ± standard deviation (*n* = 3 per group).

In the above experiments, zileuton and montelukast showed inhibitory effects on cell proliferation at concentrations of 100 and 50 µM, respectively, in both RBE and SSP-25 cell lines. To determine whether leukotriene inhibitors with different mechanisms exhibit synergistic effects when administered concurrently, we measured the cell proliferation potential of 100 µM zileuton and 50 µM montelukast administered alone or concurrently during RBE/SSP-25 incubation. In both cell lines, the co-administration of zileuton and montelukast synergistically enhanced the effects compared with each agent alone (zileuton vs. co-administration in RBE cells: 0.90 ± 0.06, vs. 0.64 ± 0.04, *p* < 0.01, in SSP-25: 0.84 ± 0.04, vs. 0.41 ± 0.03, *p* < 0.001; and montelukast vs. co-administration in RBE cells: 0.75 ± 0.04, vs. 0.64 ± 0.04, *p* < 0.01, in SSP-25: 0.69 ± 0.05, vs. 0.41 ± 0.03, *p* < 0.001) (Figure 5A).

To investigate the effects of zileuton and montelukast on apoptosis, we used the annexin V-fluorescein isothiocyanate/propidium iodide (FITC/PI) staining method with flow cytometry. Interestingly, there was no significant difference in the percentage of apoptotic cells between the control and treatment groups (100 µM zileuton and/or 50 µM montelukast) in RBE or SSP-25 cells after 72 h of treatment (Figure 5B). Next, we explored the effect of zileuton and montelukast on the cell cycle using flow cytometry (fluorescence-activated cell sorting [FACS]). RBE and SSP-25 cells treated with 100 µM zileuton or 50 µM montelukast led to greater accumulation of cells in the G0/G1 phase compared with the control (control vs. zileuton in RBE cells: G0/G1 phase rate: 43.4 ± 0.73% vs. 50.9 ± 1.49%, *p*  <  0.01, in SSP-25 cells: G0/G1 phase rate: 46.7 ± 1.13% vs. 52.9 ± 2.24%, *p*  <  0.05, and control vs. montelukast in RBE cells: G0/G1 phase rate: 43.4 ± 0.73% vs. 49.5 ± 2.55%, *p*  <  0.05; and in SSP-25 cells: G0/G1 phase rate: 46.7 ± 1.13% vs. 52.5 ± 0.25%, *p*  <  0.01)). Additionally, cells treated with the combination of 100 µM zileuton and/or 50 µM montelukast showed a significant increase in the G0/G1 phase compared with cells treated with either agent alone (zileuton vs. co-administration in RBE cells: G0/G1 phase rate: 50.9 ± 1.49% vs. 59.2 ± 1.11%, *p*  <  0.01, in SSP-25 cells: G0/G1 phase rate: 52.9 ± 2.24% vs. 56.8 ± 1.48%, *p*  <  0.05; and montelukast vs. co-administration in RBE cells: G0/G1 phase rate: 49.5 ± 2.55% vs. 59.2 ± 1.11%, *p*  <  0.01, in SSP-25 cells: G0/G1 phase rate: 52.5 ± 0.25% vs. 56.8 ± 1.48%, *p*  <  0.01) (Figure 5C).

## 3. Discussion

CCA is a hepatic malignancy that accounts for 10–20% of newly diagnosed liver cancers and is characterized by biliary tract differentiation [1,2]. Unfortunately, most cases of CCA are diagnosed at an advanced stage and require systemic chemotherapy rather than surgery [2]. However, conventional gemcitabine-based chemotherapy strategies are often ineffective due to the early development of drug resistance [3]. The efficacy of newer therapies such as immune checkpoint inhibitors and molecularly targeted drugs has recently been reported, but problems remain, including treatment limitations due to comorbidities and characteristic side effects (e.g., irAE) that are sometimes fatal [4]. Therefore, the development of new treatment approaches for CCA is urgently needed.

DR, the investigation of existing drugs for new therapeutic purposes, is one of the research tools with the most potential to identify new prophylactic and therapeutic agents [5]. DR can provide effective drugs quickly and at low cost because the compounds have already undergone clinical trials and have been shown to have no safety or pharmacokinetic issues. The development of cancer drugs is typically long and costly, making DR a powerful alternative strategy for finding effective treatments among existing drugs [6,7]. There are scattered reports of DR in cancer, such as the improved clinical outcomes of prostate and pancreatic cancer associated with angiotensin II receptor blockers [8,9] and the improved survival of patients with breast cancer using H1 antihistamines [10]. In reviewing previous reports, we focused on the observation that patients taking LTRAs for asthma had lower incidences of lung, colorectal, and liver cancer [11]. Although CCA was not included in that analysis, we designed the present study to investigate the effect of leukotrienes on biliary tract cancers; these originate in the biliary tract, which is embryologically similar to the liver.

Leukotrienes are a group of inflammatory lipid mediators synthesized from arachidonic acid. Free arachidonic acid, released from cell membranes by irritation or injury, is dehydrated and degraded by the key enzyme 5-LO, which converts it into leukotriene C_4_ (LTC_4_) via several steps. LTC_4_ is then transported extracellularly and converted to LTD_4_ and leukotriene E_4_ (LTE_4_). LTC_4_, LTD_4_, and LTE_4_ are collectively referred to as CysLTs because of their similar actions and structural features [13]. There are three receptors for CysLTs: CysLTR1–CysLTR3. Montelukast, which specifically targets CysLTR1, is one of the most important LTRAs currently in clinical use. Additionally, zileuton, a 5-LOI, is the only agent in clinical use that inhibits leukotriene synthesis. 5-LOIs reduce the levels of all CysLTs by inhibiting the first step of leukotriene synthesis, thereby reducing receptor stimulation. In our study, we performed experiments with these two anti-leukotriene drugs, which have different mechanisms of action, as well as with LTD_4_, the ligand with the strongest affinity for CysLTR1.

Our experiments with clinical samples showed that bile contains significantly higher concentrations of CysLTs than serum. This is the first report of CysLT concentrations in both bile and serum from the same patient. Because it is challenging to obtain bile from healthy individuals in a minimally invasive way, we used asymptomatic patients with bile duct stones as healthy controls. Next, we determined CysLTR1 overexpression in cancerous areas of CCA surgical specimens. High expression of CysLTR1 has been reported in pancreatic, colorectal, gastric, and breast cancers, with some studies linking high expression levels to reduced survival of patients with cancer [14,15,16,17,18]. Although our study included only 10 cases and we could not confirm a relationship between expression levels and prognosis, it is possible that CCAs are more susceptible to leukotriene-induced effects on cell proliferation than are other carcinomas. This susceptibility could be due to the expression of CysLTR1 in CCA cells and their constant exposure to high levels of CysLTs in bile.

In the in vitro experiments, the addition of LTD_4_ to CCA cell lines expressing CysLTR1 (RBE and SSP-25) promoted proliferation. However, LTD_4_ did not alter the migratory capacity of these cells. This is the first report confirming the effect of LTD_4_ on CCA cell lines. Intracellular signaling changes included enhanced phosphorylation in the MAPK and the PI3K/AKT/mTOR cascades. Activation of CysLTR1 by LTD_4_ induces phosphorylation of the MAPK cascade in mesangial cells, airway smooth muscle cells, and human mast cells [19], and it has been found to affect cancer growth through a similar pathway in pancreatic and colon cancer [14,20]. Additionally, some reports have indicated that leukotrienes affect the PI3K/AKT/mTOR cascade when exerting their biological activity [21], suggesting that multiple pathways are involved in the regulation of cancer cell proliferation.

In CCA cell line inhibition experiments with anti-leukotriene drugs, both montelukast (an LTRA) and zileuton (a 5-LOI) showed concentration-dependent inhibition of cell growth and reduced cell migration capacity. In terms of cell signaling, both drugs inhibited the MAPK and PI3K/AKT/mTOR cascades relative to ligand stimulation. Montelukast has previously been reported to inhibit cell proliferation in a dose-dependent manner in pancreatic cancer, lung cancer, and urological malignancies [14,22,23,24], which is consistent with the results of the present study. Few studies have investigated the anti-cancer effects of zileuton in vitro, with only one report of inhibition of proliferation and migration in a CCA cell line different from those used in the present experiments [25]. In vivo, zileuton has been shown to prevent lung carcinogenesis [26]. Cell cycle analysis showed that the inhibitory effect of montelukast and zileuton on cell proliferation was due to G0/G1 arrest of the cell cycle, without inducing apoptosis. The inhibition of cell proliferation by montelukast administration has been reported to be mediated by apoptosis [22,23,24,27], induction of G0/G1 arrest of the cell cycle [14], or both effects [28]. Depending on the type of cancer, it has been suggested that CysLTs may control growth through different signaling pathways and cell proliferation mechanisms.

The most interesting result of the experiments in this study was the enhanced efficacy observed with the simultaneous administration of clinical drugs with different mechanisms of action: montelukast (an LTRA) and zileuton (a 5-LOI). Montelukast is an antagonist that competitively inhibits only CysLTR1 and has previously been reported to reduce the risk of lung, colon, liver, and breast cancer in a dose-dependent manner when taken orally for the treatment of asthma [13].

There are two possible mechanisms by which zileuton may enhance this effect. First, reduced synthesis of LTD_4_ may enhance the competitive inhibitory effect on CysLTR1. Second, the effect of CysLTs on CysLTR2 and CysLTR3, in addition to CysLTR1, may be reduced, amplifying the overall inhibitory effect. Many previous reports have indicated that the stimulation of CysLTR2 does not promote cell proliferation; rather, there have been scattered reports that a low expression of CysLTR1 and high expression of CysLTR2 are associated with a good prognosis [18,29,30]. These reports suggest that decreased synthesis of LTD_4_ enhances the competitive inhibitory effect of montelukast on CysLTR1.

In this experiment, the autocrine/paracrine action of LTD_4_ synthesized and released by RBE and SSP-25 cells regulated cell proliferation in vitro. However, in vivo, other cells such as leukocytes and macrophages synthesize and release more CysLTs. The extent of the inhibitory effect in in vivo experiments needs to be confirmed in further studies to demonstrate the true value of this co-dosing enhancement.

Anti-leukotriene drugs, already in clinical use in many countries, have multiple anti-inflammatory actions in vivo. The present experimental results suggest that these drugs may inhibit several important pathways involved in the progression of biliary tract cancer. Consequently, their anti-cancer effects may attract attention in various fields.

## 4. Materials and Methods

### 4.1. Clinical Samples (Bile and Serum)

From June 2019 to June 2022, bile and serum samples were collected from patients with common bile duct stones at Nagoya City University Hospital. In total, 21 patients were enrolled, and 8 patients without inflammation (white blood cell count < 10,000/µL and C-reactive protein concentration < 2.0 mg/dL) were selected for our study. Bile samples were obtained via endoscopic retrograde cholangiopancreatography. Bile samples (5–10 mL) were aspirated after cannulation of the bile duct without contrast agent injection. Following collection, the samples were centrifuged at 2000× *g* for 10 min to remove debris. One milliliter of the collected bile samples was used for analysis. Peripheral blood was collected for serum samples the day after bile sampling. Serum was separated by centrifugation at 1500× *g* for 5 min. The purified samples were stored at −80 °C until use. The clinical characteristics of the patients included in the experiments are summarized in Supplementary Table S1.

### 4.2. Clinical Samples (Pathological Specimens)

Pathological specimens were obtained from patients with intrahepatic CCA who underwent surgery between April 2008 and October 2010 at Nagoya City University Hospital (n = 10). Formalin-fixed, paraffin-embedded samples were sectioned (4 µm) and stained using CysLTR1 antibodies (Abcam, Cambridge, UK) at 100-fold dilution. High-spot areas were captured under a fluorescence microscope (BZ-X810; KEYENCE Co., Ltd., Osaka, Japan). The clinical characteristics of the patients included the experiments are summarized in Supplementary Table S2.

### 4.3. ELISA

The CysLT concentrations in bile and serum were measured using the CysLT Express EIA Kit (Cayman Chemicals, Ann Arbor, MI, USA) without diluting the samples in accordance with the manufacturer’s protocol.

### 4.4. Cell Culture

The human intrahepatic CCA cell lines HuCCT-1, RBE, and SSP-25 were obtained from the RIKEN BRC Cell Bank (Tsukuba, Japan). The human immortalized cholangiocyte cell line MMNK-1 was obtained from the JCRB Cell Bank (Osaka, Japan). HuCCT-1, RBE, and SSP-25 cells were maintained in RPMI 1640 medium (Wako Pure Chemical Industries Co., Ltd., Osaka, Japan) supplemented with or without 10% fetal bovine serum in an incubator with 5% carbon dioxide at 37 °C. MMNK-1 cells were maintained in Dulbecco’s modified Eagle’s medium (DMEM) (Wako Pure Chemical Industries Co., Ltd.) with or without 5% fetal bovine serum under the same conditions.

### 4.5. Western Blot Analysis

The cells were lysed in lysis buffer, and 20 µL of the protein lysate sample was fractionated on polyacrylamide gels (TGX™ FastCast™ Acrylamide Kit; Bio-Rad Laboratories, Hercules, CA, USA) and then electroblotted onto nitrocellulose membranes. The membranes were blocked with 5% skim milk in phosphate-buffered saline-Tween 20 (PBS-T). They were incubated with primary antibodies followed by secondary antibodies. Enhanced chemiluminescence detection reagents (Amersham™; Cytiva, Marlborough, MA, USA) were then applied, and chemiluminescent signals were visualized as bands using an LAS 4000 mini analyzer (Cytiva). Antibodies to CysLTR1 were purchased from Abcam, and antibodies to p-AKT (Ser473), AKT, p-ERK1/2, and ERK1/2 were obtained from Cell Signaling Technology (Danvers, MA, USA). A monoclonal beta-actin antibody (FUJIFILM Wako Pure Chemical Corp., Tokyo, Japan) was used as an internal control. These investigations were independently performed on three pairs of samples.

### 4.6. Cell Viability Assays

Cell viability was measured using Cell Counting Kit-8 assay (Dojindo, Kumamoto, Japan) and evaluated according to the absorption of WST-1. The cells were seeded at a density of 3.0 × 10^3^ cells/well in 96-well plates. After overnight incubation, the cells were treated with or without different concentrations of LTD_4_ (50 nM) (Cayman Chemicals), zileuton (50, 75, 100, 125, and 150 µM) (Sigma-Aldrich, St. Louis, MO, USA), and montelukast sodium (30, 40, 50, 60, and 70 µM) (Tokyo Chemical Industry, Tokyo, Japan) for 72 h.

### 4.7. Wound-Healing Assay (Scratch Assay)

The cells were grown to confluence in 12-well plates, and then a straight wound was made using a sterile 200 µL pipette tip. LTD_4_ (50 nM), zileuton (100 µM), or montelukast (50 µM) was then added to the cells. The straight wound was photographed and measured under a microscope at 0, 12, 24, and 48 h. These investigations were independently performed on three pairs of samples.

### 4.8. Flow Cytometry Analysis

The cells were seeded in Petri dishes (1.0 × 10^5^ per well) and cultured overnight. They were then treated with or without zileuton (100 µmol/L) and/or montelukast (50 µmol/L) for 72 h. After treatment, both floating and attached cells were collected and stained. Flow cytometric analysis was performed using a flow cytometer (FACS Canto II; BD Biosciences, Franklin Lakes, NJ, USA). Cell cycles were evaluated by PI staining (PI Solution; Dojindo), and apoptosis was detected using the Annexin-V Cell Apoptosis Detection Kit 1 (BD Biosciences) according to the manufacturer’s instructions.

### 4.9. Statistical Analysis

The data were analyzed using Student’s *t*-test and the Mann–Whitney U test. Differences were considered statistically significant at *p* < 0.05. Data are expressed as mean ± standard deviation.

## 5. Conclusions

Our findings suggest that montelukast and zileuton suppressed cell proliferation in CCA cell lines by inhibiting non-apoptotic pathways. Widely used anti-allergy drugs may be useful as novel therapeutic agents for biliary tract cancer in the future.

## Data Availability

The data presented in this study are available upon request from the corresponding author.

## Supplementary Materials

The following supporting information can be downloaded at: https://www.mdpi.com/article/10.3390/molecules29143379/s1, Figure S1–S4: Whole Western blots (uncropped images) showing all bands with molecular weight markers, Figure S5: Cell cycle analysis of RBE and SSP-25 treated with zileuton and/or montelukast, Table S1: The characteristics of the patients selected in bile and serum experiments, Table S2: The characteristics of patients diagnosed and operated on for intrahepatic cholangiocarcinoma, Table S3: Antibodies and dilutions for immunoblotting.
