# Supplementary material for: Potential of Anti-Leukotriene Drugs as New Therapeutic Agents for Inhibiting Cholangiocarcinoma Progression
Source: Molecules. 2024 Jul 18;29(14):3379. doi: 10.3390/molecules29143379 (PMC11280175; doi:10.3390/molecules29143379)
Supplement: Supplementary file 1 [file molecules-29-03379-s001.zip › molecules-3091807-supplementary.pdf]

Supplementary Materials

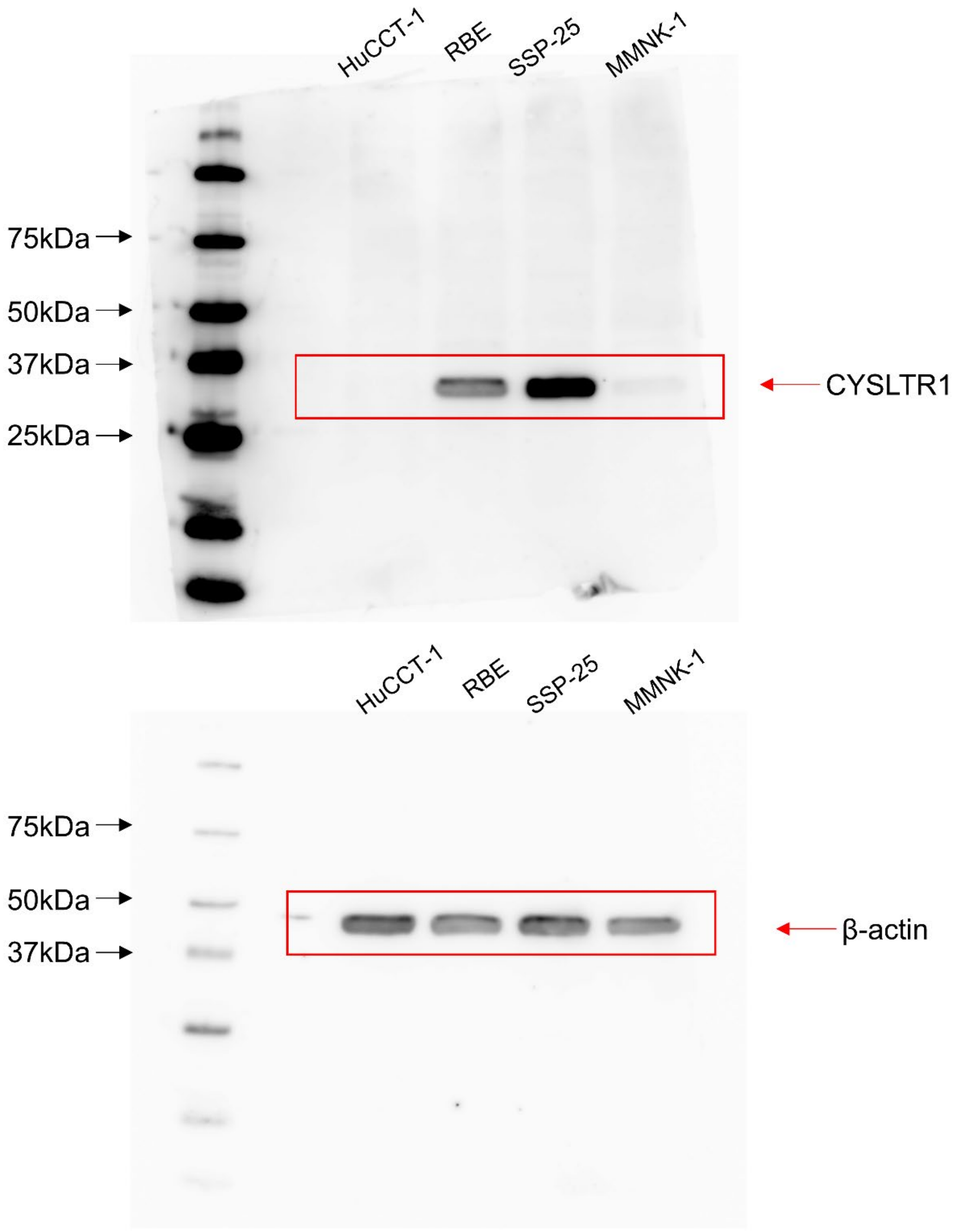

Figure S1. Whole western blots (uncropped images) showing all bands with molecular weight markers. These gels correspond to those shown in Figure 1C.

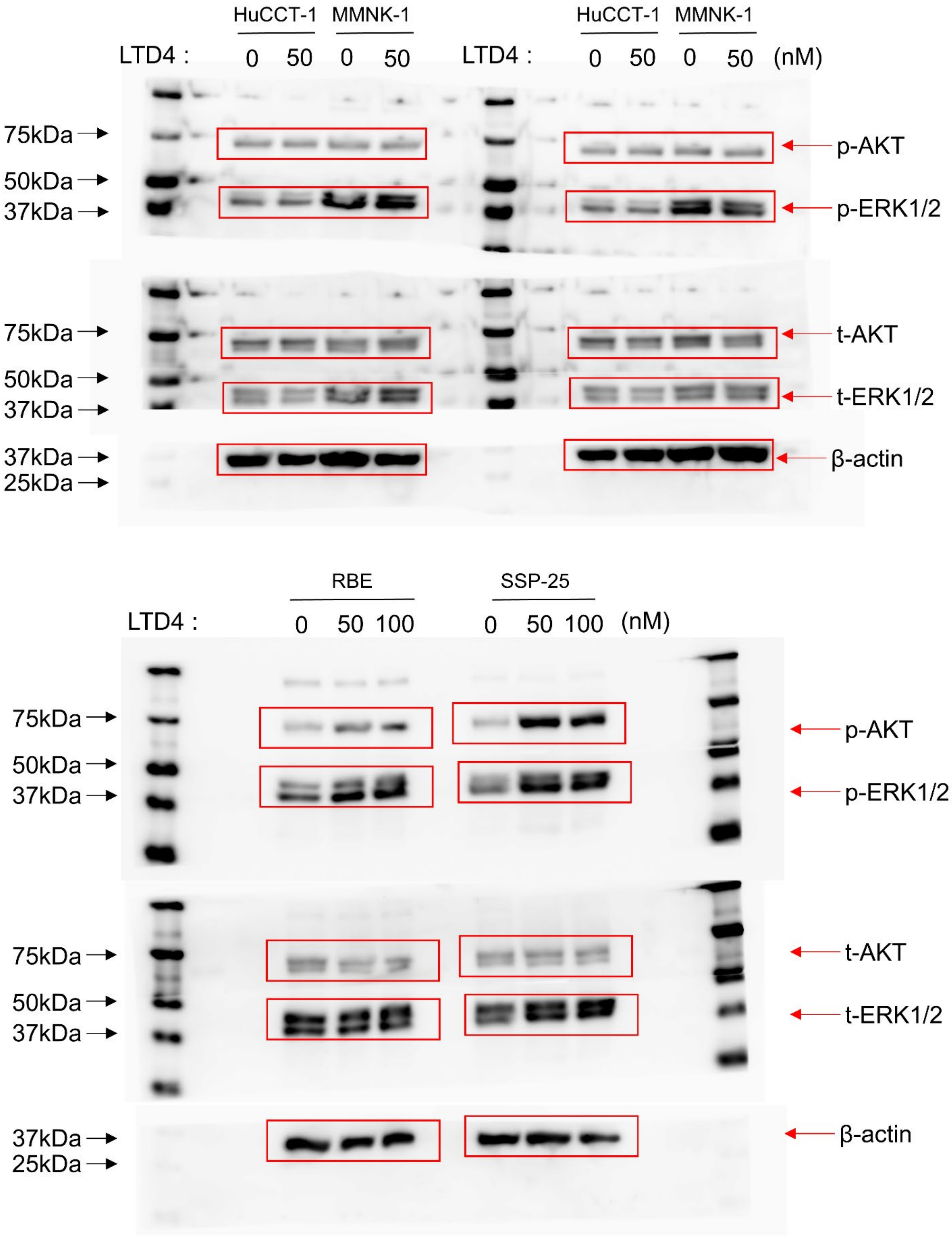

Figure S2. Whole western blots (uncropped images) showing all bands with molecular weight markers. These gels correspond to those shown in Figure 2B.

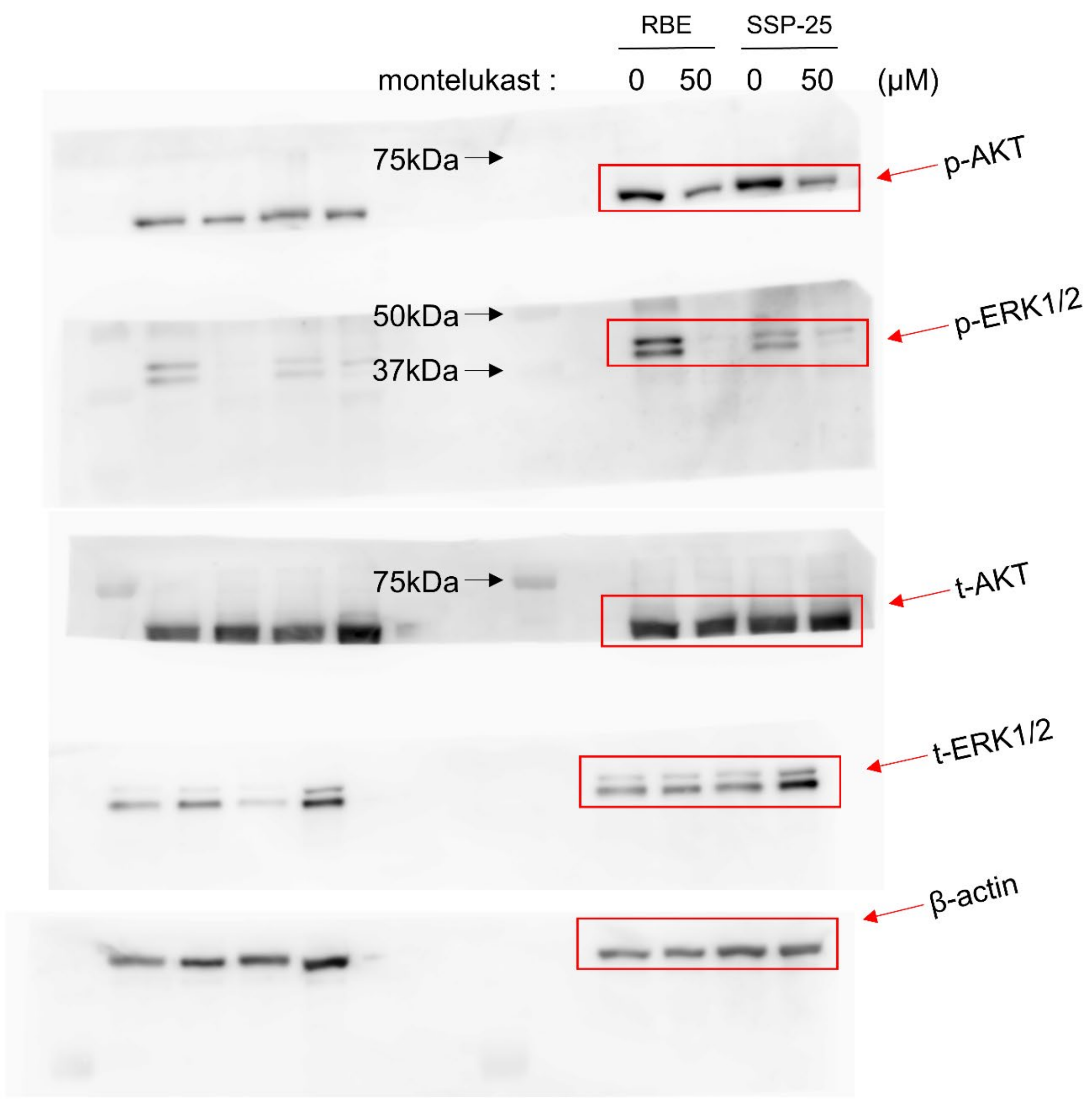

Figure S3. Whole western blots (uncropped images) showing all bands with molecular weight markers. These gels correspond to those shown in Figure 3B.

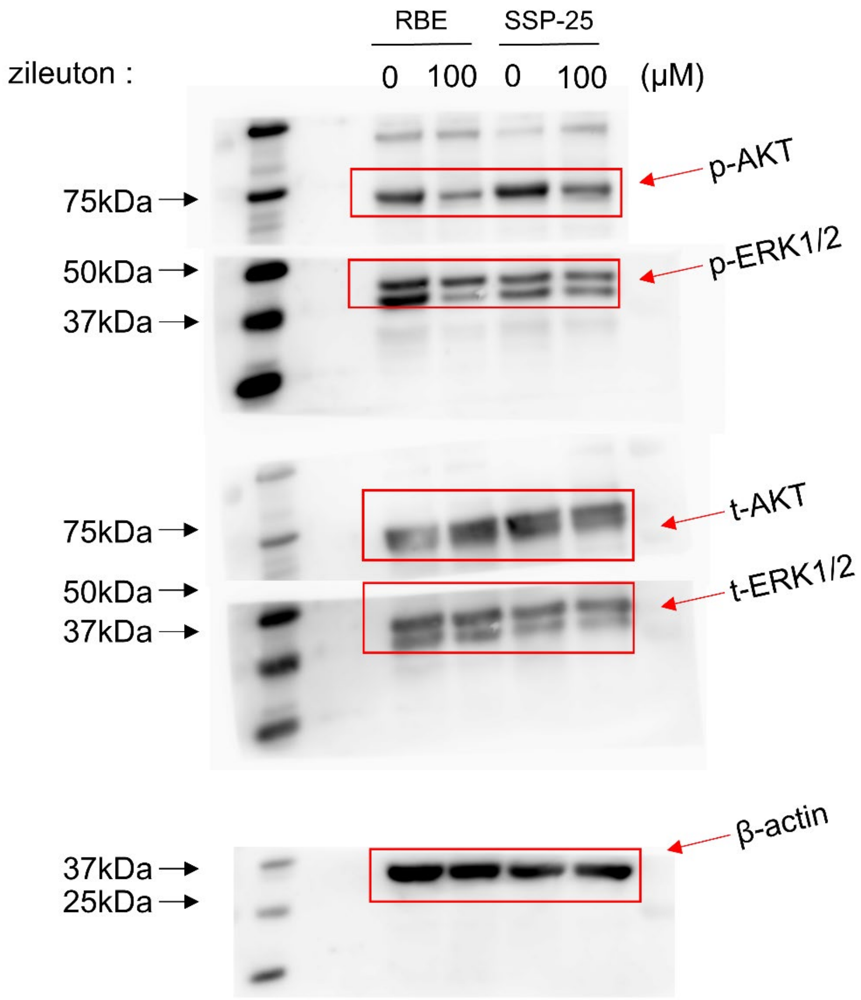

Figure S4. Whole western blots (uncropped images) showing all bands with molecular weight markers. These gels correspond to those shown in Figure 4C.

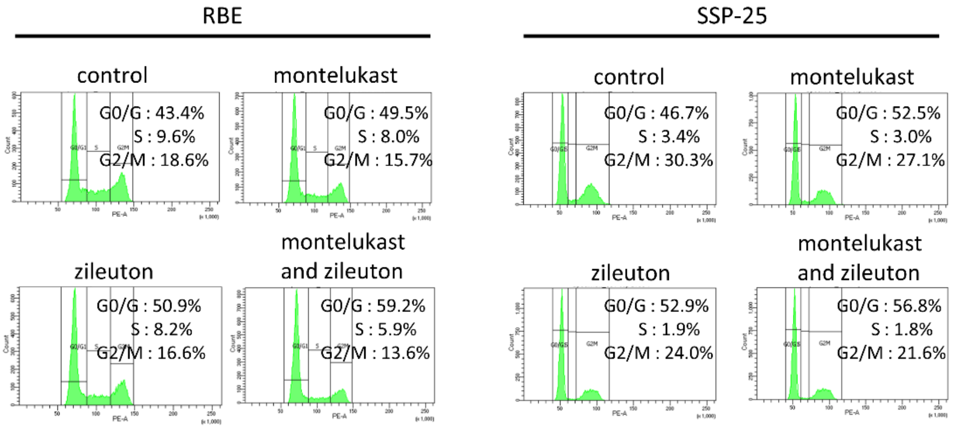

Figure S5. Cell cycle analysis of RBE and SSP-25 treated with montelukast and/or zileuton.

Table S1. The characteristics of the patients selected in bile and serum experiments.

No.	Age	Sex	anti-LT drug	WBC	CRP	Bil	ALP	γ-GT	AST	ALT	AMY
1	64	m	-	5500	0.05	0.6	338	286	22	52	86
2	64	m	-	4500	0.96	0.8	723	549	295	382	198
3	51	f	-	3200	0.42	2.5	787	868	123	342	76
4	73	f	-	3800	1.99	2.7	1140	245	115	247	33
5	65	m	-	5500	0.5	0.7	282	103	26	35	81
6	81	m	-	3700	1.67	4	638	612	147	196	55
7	71	f	-	5500	1.47	0.8	632	112	35	76	50
8	53	m	-	7500	0.33	3.7	206	752	342	377	96

Table S2. The Characteristics of patients diagnosed and operated on for intrahepatic cholangiocarcinoma.

No.	Age	Histology	Pathological stage (UICC-8)	Immunostaining
1	70	well	III	+
2	81	well-mod	III	+
3	74	well-mod	III	-
4	76	well	IVa	+
5	65	mod	I	+
6	81	mod-por	III	+
7	74	well	III	-
8	62	por	IVa	-
9	51	well	I	+
10	61	well	I	+

Table S3. Antibodies and dilutions for immunoblotting.

Protein	Dilution	Antibodies
β-Actin	5000	FUJIFILM Wako Pure Chemical Corp
CYSLTR1	1000	Abcam plc. (Cambridge, UK)
AKT	1000	Cell Signaling Technology, Inc. (Danvers, MA, USA)
Phospho-AKT	1000	Cell Signaling Technology, Inc. (Danvers, MA, USA)
ERK1/2	2000	Cell Signaling Technology, Inc. (Danvers, MA, USA)
Phospho-ERK1/2	2000	Cell Signaling Technology, Inc. (Danvers, MA, USA)
